# When the Seasons Don't Fit: Speedy Molt as a Routine Carry-Over Cost of Reproduction

**DOI:** 10.1371/journal.pone.0053890

**Published:** 2013-01-17

**Authors:** Maurine W. Dietz, Ken G. Rogers, Theunis Piersma

**Affiliations:** 1 Animal Ecology Group, Centre for Ecological and Evolutionary Studies, University of Groningen, Groningen, The Netherlands; 2 St. Andrews, Victoria, Australia; 3 Department of Marine Ecology, Royal Netherlands Institute for Sea Research, Den Burg, Texel, The Netherlands; CNRS, Université de Bourgogne, France

## Abstract

The failure of animals to fit all life-cycle stages into an annual cycle could reduce the chances of successful breeding. In some cases, non-optimal strategies will be adopted in order to maintain the life-cycle within the scope of one year. We studied trade-offs made by a High Arctic migrant shorebird, the red knot *Calidris canutus islandica*, between reproduction and wing feather molt carried out in the non-breeding period in the Dutch Wadden Sea. We compared primary molt duration between birds undertaking the full migratory and breeding schedule with birds that forego breeding because they are young or are maintained in captivity. Molt duration was ca. 71 days in breeding adults, which was achieved by an accelerated feather replacement strategy. Second-year birds and captive adults took ca. 22% and 27% longer, respectively. Second-year birds start molt in late June, more than four weeks before captive adults, and almost seven weeks before adults that return from breeding in late July–August. Adults finish molt in October when steeply increasing thermostatic costs and reductions in food availability occur. Primary molt duration was longer in female than in male knots (all ages), which was accordance with the somewhat larger body size of females. Since fast growth leads to lower quality feathers, the speedy wing molt shown by Arctic-breeding birds may represent a time constraint that is an unavoidable and routine cost of reproduction. So far it was hypothesized that only birds over 1 kg would have difficulty fitting molt within a year. Here we show that in birds an order of magnitude smaller, temporal imperatives may impose the adoption of non-optimal life-cycle routines in the entire actively breeding population.

## Introduction

Organisms have developed an endless variety of strategies to exploit what the most productive seasons offer and to cope with what the harshest seasons dictate. Relatively mobile animals have opportunities to use resources and avoid environmental stressors by strategically moving across the seasonally changing landscapes [1], [2]. Nevertheless, there is one hard boundary condition for all: the length of the year [3]–[5]. Whether sedentary or migratory, to cope with the seasonal changing environments, adult vertebrates each year go through several consecutive life-cycle stages during which they adjust their morphology, physiology and behavior [6], [7]. To maximize reproductive opportunities, animals will generally try to complete all necessary seasonal activities within the period of a year [5]. Small species with shorter life-cycle stages, or animals with fewer such stages per year, are predicted to be relatively flexible in the timing of these stages [4], [7]. For some animals, particularly larger ones, it will take longer to complete a full cycle of life-history stages. This may also apply when mutually exclusive activities, such as breeding and migration, are necessary or when maintenance of plumage or pelage (e.g. flight feather molt) requires dedicated time [4].

Some bird species carry out molt at the same time as other life-cycle stages [7], [8]. Molt can also be interrupted and spread over a longer time [9], and the rates and extent of molt can be adjusted to individual circumstances [10], [11]. Birds that accelerate molt incur costs in terms of increased daily energy costs for molt, flight and thermoregulation, decreased flight abilities and therefore increased predation danger, and a lower quality of feathers grown, resulting in negative effects on e.g. pigmentation or ornaments, length, resistance to wear, and insulation [10], [12]–[21]. In long-distance migrant shorebirds, i.e. habitat specialists with tightly timed annual cycles [22], [23], wing molt rarely overlaps with breeding, and even more rarely with migration [12], [24]. North temperate wintering shorebirds have to complete molt before the onset of severe weather conditions in late autumn [12], [25] when they may be faced with time constraints on the completion of molt.

To examine possible trade-offs between breeding and migration, and wing molt, we investigated timing and duration of primary molt in red knots *Calidris canutus islandica* in comparative and experimental ways. These birds breed on the High Arctic tundra in Greenland and Canada, and molt and winter in the Dutch Wadden Sea, the UK, and the French Atlantic coast [26]. Our study focused on red knots wintering in the Dutch Wadden Sea. We compared their performance with those of birds not spending time on breeding and migration. These include second-year birds that remain in the Wadden Sea the whole summer [24], [27] and birds kept in captivity in outdoor aviaries. The latter two categories were not constrained in time or food, and served as a reference point against which actual molt of possibly time-pressed reproducing adults were compared.

## Materials and Methods

### Ethics Statement

All handling of the birds complied with the Dutch Flora and Fauna Law and the Dutch Law on Animal Experiments, and was covered by the permit DEC-NIOZ-08.01 issued by the Animal Experiments Committee of the Royal Netherlands Academy of Sciences (DEC-KNAW). Mist netting of the red knots was supervised by very experienced bird catchers, who also ringed the birds and measured their biometry and scored molt. The birds were released as soon as possible. Molt scoring of the captive Red Knots was part of the caretaking routine and did therefore not impose any extra discomfort on the animals. The red knots were brought into captivity for other purposes several years earlier.

### Life Cycle Stages of Red Knot *Calidris canutus islandica*

In June, red knots (subspecies *islandica*) breed at the arctic tundra of Greenland and Canada. Both parents incubate the eggs, but after hatching, the females leave to prepare for migration to the wintering grounds in the Dutch Wadden Sea, the UK, and the French Atlantic coast [26], [28]–[31]. The male cares for the young. When the young are independent, the males depart to wintering grounds [31]. The young depart later and migrate independent of the adults. Due to this asynchrony, and because most but probably not all birds fly directly to the wintering areas without a stopover [27], the arrival period at the wintering grounds is long (July–September), with first the adult females, then the adult males and lastly the young arriving [31], [32]. Adults start molting flight and body feathers shortly after arrival [23], [25], [27], [33]; the juveniles do not molt flight feathers. At the end of winter, a second period of body molt starts resulting in a full rufous breeding plumage in adults in early May [25]. During this body molt, adults prepare for spring migration in early April, and fly the first leg of migration at the end of April [32]. After a stopover at Iceland or northern Norway, the knots reach their breeding grounds in late May/early June [28], [32]. Second-years, i.e. birds born in the year before, remain in the wintering area during summer [25], [27]. However, we cannot exclude that a few second-years may migrate northwards as far as the staging areas (to the best of our knowledge no second-year birds were ever recorded in the breeding areas), nor can we exclude that a few adults may oversummer in the Wadden Sea e.g. due to poor condition.

### Free-living Birds

In 1998–2006, red knots were caught with mist nets close to a high tide roost in the Dutch Wadden Sea (Richel, 53°16′N 5°08′E, or Simonszand, 53°31′N 6°23′E) during New Moon periods (dark nights) in July–October. After capture, they were banded and body mass (±1 g), general biometry and feather growth scores determined. Primary growth was scored in the left wing from 0 (old primary) to 5 (new primary) conforming with [9]. The sum of the feather growth scores over all primaries gives the primary molt score. Age (juvenile, second-year, or adult) was determined from plumage characteristics [34]. A small blood sample was drawn from the wing vein and stored in 95% ethanol for molecular sex determination [35].

From mid-July onward, two subspecies of red knot occur in the Dutch Wadden Sea: *C. c. islandica* which overwinters and molts there, and *C. c. canutus* which fuels up to migrate further to west Africa where they overwinter [28], [36]. Although the two subspecies differ in body size [29], [37], the large overlap precludes the use of body size to distinguish subspecies. However, as second-year and adult *islandica* knots molt flight feathers on the Wadden Sea [25], [32] and *canutus* knots do not, birds in active primary molt were assumed to be of *C. c. islandica*. There were a few outliers with atypical molt patterns and suspended molt in the data set (22 adult males, 26 adult females, 4 second-year males and 4 second-year females). Including the outliers in the analysis did not significantly change the results, but because we are interested in the typical primary molt pattern of the average red knot, we nevertheless deleted them. Final sample sizes were 522 adult males, 758 adult females, 258 second-year males, and 319 second-year females.

### Captive Birds

In 2009, 32 adult *C. c. islandica* knots (21 males and 11 females) were housed in groups of 8 birds in open outdoor aviaries at the Royal Netherlands Institute for Sea Research (NIOZ) (53°00′N 4°47′E). Red knots were fed *ad libitum* trout pellets (Trouvit Classic 2P, Skretting, Hendrix SpA, Italy; composition: crude protein 45%, carbohydrate 21%, crude fat 16%, crude ash 9%, lysin 3%, indigestible fibres 2%, phosphorus 1%). For details of the housing conditions see [38]. The birds experienced the local light-dark cycle and ambient temperatures and under these conditions *C. c. islandica* knots maintain natural seasonal cycles in body mass, molt, and physiological characteristics such as corticosterone [39], [40]. Many red knots have been kept at our facility on the trout diet for over 18 years without health or molt problems, nor have they shown changes in the annual cycles of body mass and molt. The birds were examined weekly, weighed (±1 g), and primary molt was recorded.

The knots were captured in the Dutch and German Wadden Sea in the period 1994 to 2004. To investigate if long-term captivity affected primary molt, we examined also primary molt in the first year in which they went through a complete molt cycle in captivity (usually the year after the year of capture) in relation to molt in 2009. Data on 17 of 32 knots of 2009 (13 males and 4 females) were available for this analysis.

### Primary Molt Analysis of Free-living Birds

We analyzed the primary molt pattern using the models of [41] and [42]. These give estimates of molt duration and average start date (from which average end date can be calculated) and also the standard deviation of the start date, and require an index of molt increasing linearly with time. Two of the five molt situations considered by [41] and [42] were used in this study. Type 2 applied to second-year birds as all were present in the study area before any molt started. Type 4, for situations where some birds start molt before all birds are present, applied to adults. A molt index [41] which increased linearly with time was obtained using feather growth scores and relative masses of each primary (from [43], see Tables S1, S2, S3, and S4, Fig. S1).

Female knots are heavier and larger than males (e.g. [29], [37]) resulting in more feather mass to replace [44], which may affect molt duration and timing (replacing more feather mass may take more time [44]). Therefore we checked if sexes and age classes differed in body dimensions. Because wing length may vary due to feather wear, only birds that had completed wing molt were included. Female red knots were indeed heavier and larger (except for tarsus length) than males, but wing length and other body size characteristics did not differ between age classes of the same sex (Table S5).

In some free-living adults, primary molt was relatively advanced compared to the majority of adults, while in some second-years primary molt was delayed (Fig. S3). Possibly, the advanced adults had left the breeding grounds very early, e.g. after clutch failure due to predation [45], or had not migrated but over-summered in the Wadden Sea. The delayed second-years may have migrated to the stopover site, or age was not correctly determined. Since we were interested in the general pattern, we excluded these birds from the analysis.

### Primary Molt Analysis of Captive Birds

Because molt of captive birds was scored on a weekly basis, we had to adjust procedures to obtain comparable molt model parameters. The data are of Type 2 [41], but using the model failed, possibly because sample size was relative small for this model, and because the data were organized on a weekly basis. Instead, results of linear regression of molt indices against time for actively molting birds were used to give individual estimates of molt duration, start and end date. R-squared values were high, averaging 0.987 (±0.020 SD) over the 32 individuals; samples ranged from 9 to 15 observations per individual. A comparison of the body sizes of captive and free-living birds was impossible because in captive birds measurements were not made when the molt data were gathered.

Duration of captivity had no effect on the primary molt parameters; estimated molt start and end date, and molt duration did not differ between the first molt in captivity and molt in 2009 (Table S6, sexes pooled, ANOVA, F_1,47_ = 1.035, P = 0.314, and F_1,47_ = 0.028, P = 0.869, for duration and start date, respectively). The data on first molt and 2009 molt were not pooled to avoid problems with repeated measures in about half of the individuals. We used the 2009 group because that group gave the largest sample.

### Statistics

We used Julian day (January 1 is day 1) as time scale in the analyses and tables, but depicted calendar date in the graphs. The primary molt parameters of the free-living knots were estimated with the R package moult (available from the Comprehensive R Archive Network, CRAN, at url http://cran.r-project.org/package=moult; [46]). For free-living knots, we tested for differences between age categories within sex, and between males and females within age category, by modeling a series of models that estimated a combination of the variables (duration, start date and SD of start date) separately for age category or sex and calculating AIC values. We followed this procedure because the R package moult does not allow a comparison of nested groups. The best models were selected on basis of AIC-values: best models had the lowest AIC and differed in AIC by less than 2. In addition, Akaike's weight (*w*_i_) was calculated for the models. Statistical comparisons with the captive birds were not possible because different methods were necessary to estimate molt parameters in the two groups. Molt model results are presented as means ± asymptotic standard errors for free-living birds and as means ±SE for captive birds. Individual primary model results are presented as means ± asymptotic standard errors. Other data are presented as means ±SE. An ANOVA with post hoc Tukey analysis (PASW Statistics 18.0.3) was used to determine differences between means of groups.

## Results

**Figure 1:**
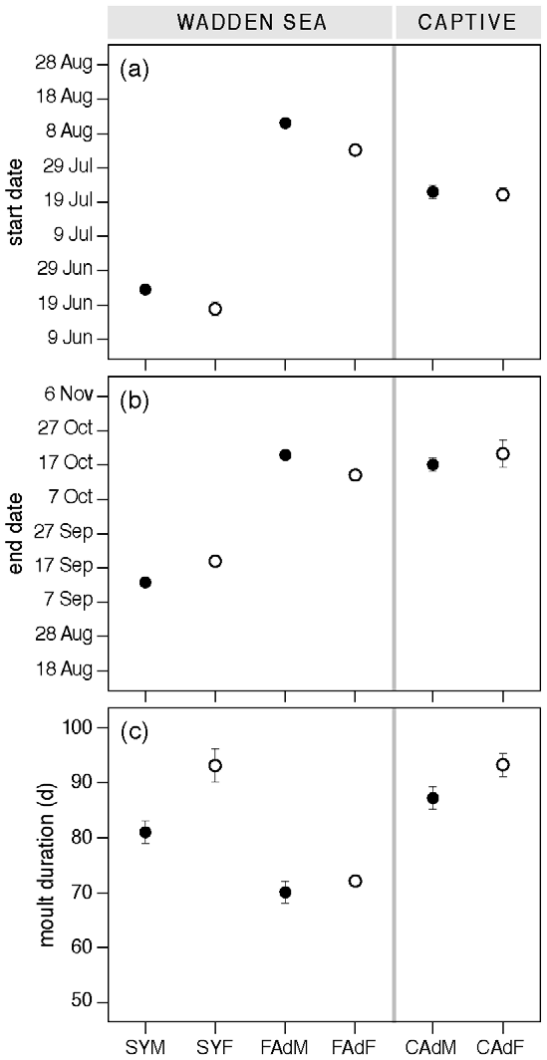
Primary molt start and end date (A, B), and molt duration (C), in free-living and captive red knots. Symbols: closed symbols, males; open symbols, females. Group abbreviations: SYM, second-year males; SYF, second-year females; FAdM, free-living adult males; FAdF, free-living adult females; CAdM, captive adult males; CAdF, captive adult females.

We predicted that wing molt in breeding adults would be time-constrained due to a trade-off between breeding and migration. The nonbreeding over-summering second-years and captive adults, not faced with this trade-off, would be predicted to use more time to molt than the reproductively active adults. Consistent with these expectations wing molt duration was shortest in free-living adults, ca. 71 days, and longer –and mutually comparable– in second-years and captive adults, varying from 81 to 93 days (Fig. 1C). For both free-living males and females, the best models were the models that estimated all molt parameters separately per age class, as indicated by the lowest AIC for these models (Table 1). Hence molt duration differed significantly between adults and second-years. Molt duration was ca. 22% longer in second-years than in free-living adults, and ca. 27% longer in captive adults. In the Supporting Information is shown how the differences in molt duration were established by presenting the individual primary molt durations for all groups (Fig. S2).

**Table 1:** Comparison of molt parameters between ages (within sex) or sex (within age) in free-living adult and second-year red knots.

Model	Δ AIC_i_	*w*_i_
*free-living males*		
duration, start date, and SD start date separately	0.0	1.0
start date and SD start date separately	11.7	0.0
duration separately	539.0	0.0
adults and second-years combined	1084.2	0.0
*free-living females*		
duration, start date, and SD start date separately	0.0	1.0
start date and SD start date separately	48.0	0.0
duration separately	558.9	0.0
adult and second-years combined	1174.4	0.0
*free-living adults*		
start date and SD start date separately	0.0	0.63
duration, start date, and SD start date separately	1.1	0.37
duration separately	78.2	0.00
males and females combined	142.8	0.00
*free-living second-years*		
duration, start date, and SD start date separately	0.0	0.99
start date and SD start date separately	9.5	0.01
duration separately	27.1	0.00
males and females combined	29.4	0.00

Best models (underlined) were selected using AICs. Models are equally plausible when the difference from the best model, ΔAIC_i_, is smaller than 2. *w*_i_ is the Akaike weight for model i.

As shown by the model comparison (Table 1), not only duration, but also timing of wing molt differed between groups. Second-years were the first to start molt (ca. 21 June), more than four weeks before the captive adults, and almost seven weeks before free-living adults started in early August (Fig. 1A, see Table S6 for details). Adults completed molt at comparable dates, mid- to late October, independently of living conditions (Fig. 1B). Second-years completed molt a month earlier.

Molt parameters differed between sexes in second-years as indicated by the lowest AIC for the model that estimated all parameters separately for males and females (Table 1, *w*_i_ = 0.99). In free-living adults, two models were equally plausible, one that estimated start date and SD of start date separately for the sexes and one that estimated all parameters separately (*w*_i_ = 0.63 and *w*_i_ = 0.37, respectively). The model estimating start date and SD of start date separately was 1.7 times (0.63/0.37) more likely than the model estimating all parameters separately. In free-living adults and second-years, females started molt before males and molted longer than males (Fig. 1). Although captive females also tended to take longer to molt than captive males, this difference was not significant (ANOVA, F_1,30_ = 4.014, P = 0.054). Start date did also not differ between the sexes in captive adults (ANOVA, F_1,30_ = 0.055, P = 0.817). Combining sexes we checked if molt parameters differed between aviaries, which was not the case (ANOVA, duration, start and end date, all P>0.4).

## Discussion

Primary molt duration of adult red knots in the Wadden Sea was similar to adult *C. c. islandica* in Scotland (77 days; [47]), but much shorter than in adult *C. c. canutus* knots in South Africa (95 days; [47]). Primary molt duration of second-years and captive adults resembled however more that of the *C. c. canutus* than of the Scottish free-living adult *C. c. islandica*. The sexual differences in molt parameters are in accord with body size differences and behavioral differences. Female knots are somewhat larger than males (Table S5; [29], [37]) and therefore have more feather mass to replace. A longer primary molt duration in females (Fig. 1) is thus not unexpected. As in many Arctic-breeding shorebirds, female and male knots both incubate the eggs, but only males care for the young. Adult females leave the breeding grounds earlier than males and arrive earlier in the wintering areas [28]–[30]. Stable isotope data indicate that knots start molt ca. 4 days after arrival in the Dutch Wadden Sea [27], and adult females are thus expected to start molt earlier than adult males (Fig. 1). That the average difference in start date (6 days) is smaller than the young-caring period (17–18 days; [31]) may be because of earlier departure of males that lose their clutch or brood, or where unable to find a mate [45].

**Figure 2:**
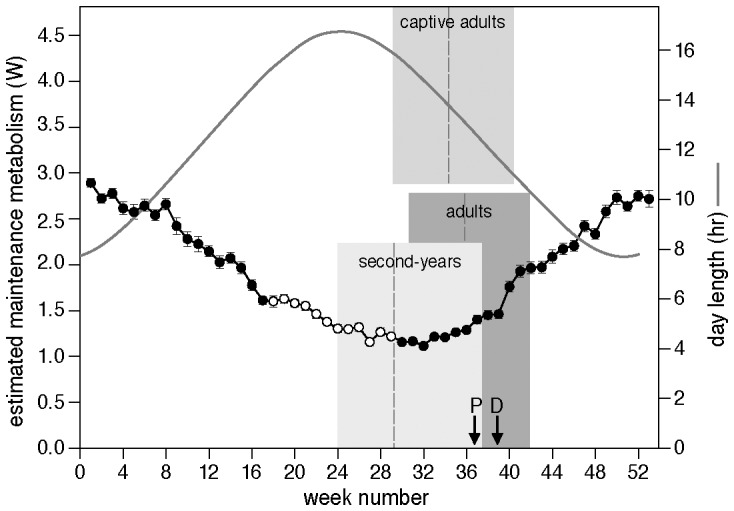
The average temporal distribution of primary molt in free-living adult and second-year red knots, and captive adult red knots (sexes combined, grey blocks). The dashed vertical grey lines indicate when the knots had completed growth of primary 5. Also plotted are the estimated weekly maintenance costs for a red knot living in the Wadden Sea, calculated using the model of [63] (equation 5; using mudflat conductances) from daily wind, ambient temperature and solar radiation data obtained over the same period as the molt data (1998–2006; data from the Royal Netherlands Meteorological Institute (KNMI) weather station at Hoorn on the island Terschelling in the Dutch Wadden Sea, 52°23′N 5°21′E). Note that basal and thermoregulatory costs are included in maintenance costs, but molt costs (feather synthesis and increased thermoregulation) are not. Maintenance costs are indicated with circles; open circles indicate the period during which adult red knots are migrating or in the breeding areas [63]. In addition, daylength in the Wadden Sea is given (solid line, right Y-axis, data from KNMI, 2006). Arrows indicate the start of the rapid increase in number of peregrines in the Wadden Sea (P; [54]), and when the diet of free-living knots changes from shellfish to less profitable mudsnails (D; [56], [58]).

Primary molt duration was much shorter (ca. 22%) in free-living adults than in the non-breeding second-years. This is also the case, with a similar percentage, in north temperate wintering grey plovers *Pluvialis squatarola* [48], which are slightly larger than red knots and have a comparable timing of molt and other life-cycle stages, including the skipping of breeding in their second year of life. Interestingly, when adult red knots were brought into captivity, molt duration increased to equal that of second-years (Fig. 1). The short molt duration in free-living adults, their ability to slow progression of molt when brought into captivity, and the rapid onset of molt after arrival in the Wadden Sea [27] suggest that molt is seriously time-constrained in free-living north-temperate wintering adult red knots. This time constraint may well relate to (1) a restriction on the onset of molt because adults do not arrive before late July in the Wadden Sea and do not molt primaries or body feathers during breeding or in transit, and (2) a restriction on the time during which molt needs to be completed because of increases in thermostatic costs and stormy weather in the course of the year. Indeed, second-years finished molt well before October, when thermoregulatory costs started to increase rapidly (Fig. 2). Free-living adults started molt when thermoregulatory costs were still low, but these showed a steep increase during the last part of molt. Precipitation, not included in the calculation of cost-levels, also increased from mid-October onwards, thus adding to the pressures later in the year. Adult molt was finished well before thermoregulatory costs had reached maximal winter levels. An important additional reason for adult knots to complete molt before thermostatic costs have increased too much is that their winter plumage provides better insulation than their summer plumage [38], [49].

There may yet be other factors. The gap in the wing during molt has negative effects on flight abilities and flight costs [50].The larger the gap, the larger this effect will be [50], and the more vulnerable will the birds be to predation [51]. The increasing precipitation, wind speed and considerably stronger wind gusts in autumn may add to the negative effects of molt on flight ability and costs. However, birds could compensate for these effects with a decrease in body mass and an increase in pectoral muscle mass [50], [52], just as captive knots decreased body mass while maintaining stable pectoral muscle mass when exposed to raptor models [53]. Free-living adult knots would have completed molt in the first five primaries before the number of peregrines *Falco peregrinus* in the Dutch Wadden Sea started to increase steeply from ca. 15 in September to ca. 45 in October ([54]; Fig. 2), and probably relied on their predation avoidance tactics during the remainder of primary molt [55]–[57]. Second-year birds were close to finishing molt at that time. Note also that the preferred and most profitable foods of knots become scarcer in September when knots tend to switch from bivalves to the less profitable mudsnail, *Hydrobia ulvea* [56], [58].

Flight feather quality is likely to be very important to a long-distance migrant that makes non-stop flights of many thousands of kilometers [23], [28]. The ubiquitous finding that fast growth, i.e. relative short molt duration, leads to lower quality feathers [13]–[21], [59]–[62] (but note that in the sedentary house sparrow, *Passer domesticus*, this effect was condition dependent [19]) that deteriorate more between molts [16]–[18], implies that the speedy wing molt carried out by northerly wintering knots represents a cost of reproduction. This trade-off may drive the timing of molt in adults in such a way that the endpoint is as late as possible, so that accumulated feather wear during northward and post-breeding southward migration is as small as possible to minimize the increase in flight costs due to feather wear. This may explain why captive adults timed molt such that the endpoint coincided with that of free-living adults and not of second-years.

That reproduction affects molt and *vice versa* has been shown before. Generally, reproductive activities tend to delay molt, e.g. resulting in reduced lean tissue mass during the pre-migratory period [59], a lower insulation [21], or a decrease in feather quality [62]. Interestingly, the carry-over effects on molt were only found in late (or experimentally delayed) breeders: i.e., some birds managed to escape the timing effect. Here we have an example where all individual northerly wintering migrating red knots are caught between a rock (the breeding season in the High Arctic and the time needed to travel there and back) and a hard place (steeply deteriorating environmental conditions in the course of autumn), simply by having a life-history that involves a migration towards and from High Arctic breeding locations. The general finding that fast growth leads to lower quality feathers implies that the speedy wing molt shown by birds that have reproduced in the High Arctic represents an unavoidable and routine cost of reproduction.

Interestingly, Hedenström [4], on the basis of a review of body size scaling relationships of different annual cycle components, predicted that only in birds reaching masses of several kilograms, a year would be too short to include breeding, molt, and migration. That large birds encounter problems with the timing of molt is also demonstrated by [45], who show that molt becomes incomplete in birds over 1 kg that maintain flight during molt. To this we can now add that even in birds an order of magnitude smaller, temporal imperatives impose the adoption of non-optimal life-cycle routines in the entire actively breeding population.

## Supporting Information

Figure S1 **The cumulative proportion of feather mass grown (PFMG) during molt in free-living adult and second-year red knots, and captive adult red knots, determined via the individual primary models.** The data were pooled for the sexes because molt models could not be fitted for primaries 1 and 7 in captive females. For second-years we had no or insufficient data for primaries 1–5 (Table S3). Since at the end of molt PFMG equals 1, the PFMG grown by primaries 1–5 could be determined and added to the proportion of feather mass grown obtained from the known primaries. For want of data, we excluded the first 10 days of available data from the graph. The thick lines show cumulative PFMG curves (solid, free-living adults; dashed, captive adults; dash-dot, second-years). The thin lines correspond to uniform growth rates. For second-years this is the estimated uniform growth rate calculated using the mean start date obtained from the general molt models and end date from the individual primary models. The horizontal grey lines indicate the quartiles of PFMG and their durations for free-living adults (continuous arrows) and captive adults (dashed arrows). In all groups PFMG increased sufficiently linearly with time to make them good indices of molt progression.
(TIF)

Figure S2 **Molt duration of individual primaries versus relative primary feather mass for free-living adult and second-year knots (solid lines) and captive adult red knots (dashed lines).** Each point on each curve corresponds to an individual primary as relative primary mass increases with increasing primary number. For adult captive females, the models did not converse to a significant solution for primaries 1 and 7. For second-years we had insufficient data of active molt for primaries 1–5 (Table S3) and data for the sexes were pooled. Closed symbols, males; open symbols, females; grey symbols, second-years; circle, Type 2 model; square, Type 4 model. The inset graph shows the number of simultaneously growing primaries (mean ± SE) for each primary in molt for the average free-living adult knot.
(TIF)

Figure S3 **The relationship between the proportion of primary feather mass grown and time of the year for free-living adult and second-year red knots, and captive adult red knots.** Left panels, males, closed symbols; right panels, females, open symbols. Solid lines represent the general molt models, dashed lines give the 95% confidence intervals.
(TIF)

Information S1 **Extra Information for Tables S1, S2, S3, and S4 and Figures S1 and S2.** Description of the Results of the Individual Primary Molt Analyses.
(DOCX)

Table S1 **Estimates (with asymptotic standard errors) of individual primary models of Types 2 and 4 for free-living adult male red knots.**
(DOCX)

Table S2 **Estimates (with asymptotic standard errors) of individual primary models of Types 2 and 4 for free-living adult female red knots.**
(DOCX)

Table S3 **Estimates (with asymptotic standard errors) of individual primary models of Types 2 and 4 for free-living second-year red knots (sexes combined).**
(DOCX)

Table S4 **Estimates (with asymptotic standard errors) of individual primary models of Types 2 and 4 for captive adult male and female red knots.**
(DOCX)

Table S5 **Body mass and body size characteristics of free-living second-year and adult red knots that had completed primary molt.**
(DOCX)

Table S6 **Final estimates (with asymptotic standard errors for free-living red knots and SE for captive knots) of general molt parameters.**
(DOCX)
