# Supplementary figures and images for: When the Seasons Don't Fit: Speedy Molt as a Routine Carry-Over Cost of Reproduction
Source: PLoS One. 2013 Jan 17;8(1):e53890. doi: 10.1371/journal.pone.0053890 (PMC3547963; doi:10.1371/journal.pone.0053890)

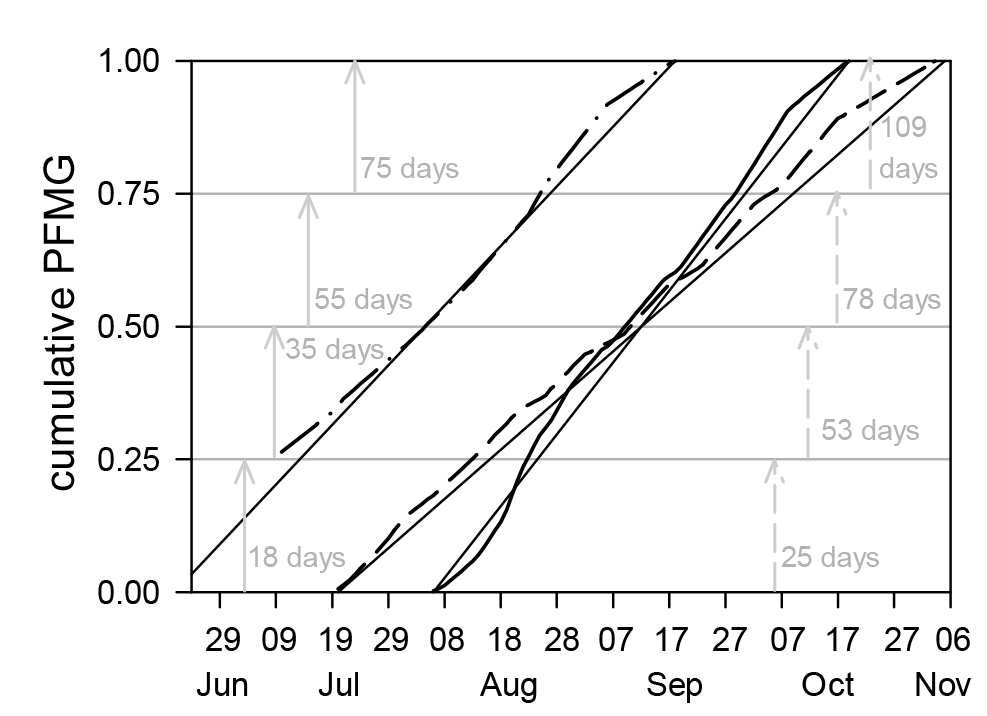

Supplement: Figure S1 — The cumulative proportion of feather mass grown (PFMG) during molt in free-living adult and second-year red knots, and captive adult red knots, determined via the individual primary models. The data were pooled for the sexes because molt models could not be fitted for primaries 1 and 7 in captive females. For second-years we had no or insufficient data for primaries 1–5 (Table S3). Since at the end of molt PFMG equals 1, the PFMG grown by primaries 1–5 could be determined and added to the proportion of feather mass grown obtained from the known primaries. For want of data, we excluded the first 10 days of available data from the graph. The thick lines show cumulative PFMG curves (solid, free-living adults; dashed, captive adults; dash-dot, second-years). The thin lines correspond to uniform growth rates. For second-years this is the estimated uniform growth rate calculated using the mean start date obtained from the general molt models and end date from the individual primary models. The horizontal grey lines indicate the quartiles of PFMG and their durations for free-living adults (continuous arrows) and captive adults (dashed arrows). In all groups PFMG increased sufficiently linearly with time to make them good indices of molt progression. (TIF) [file pone.0053890.s001.tif]

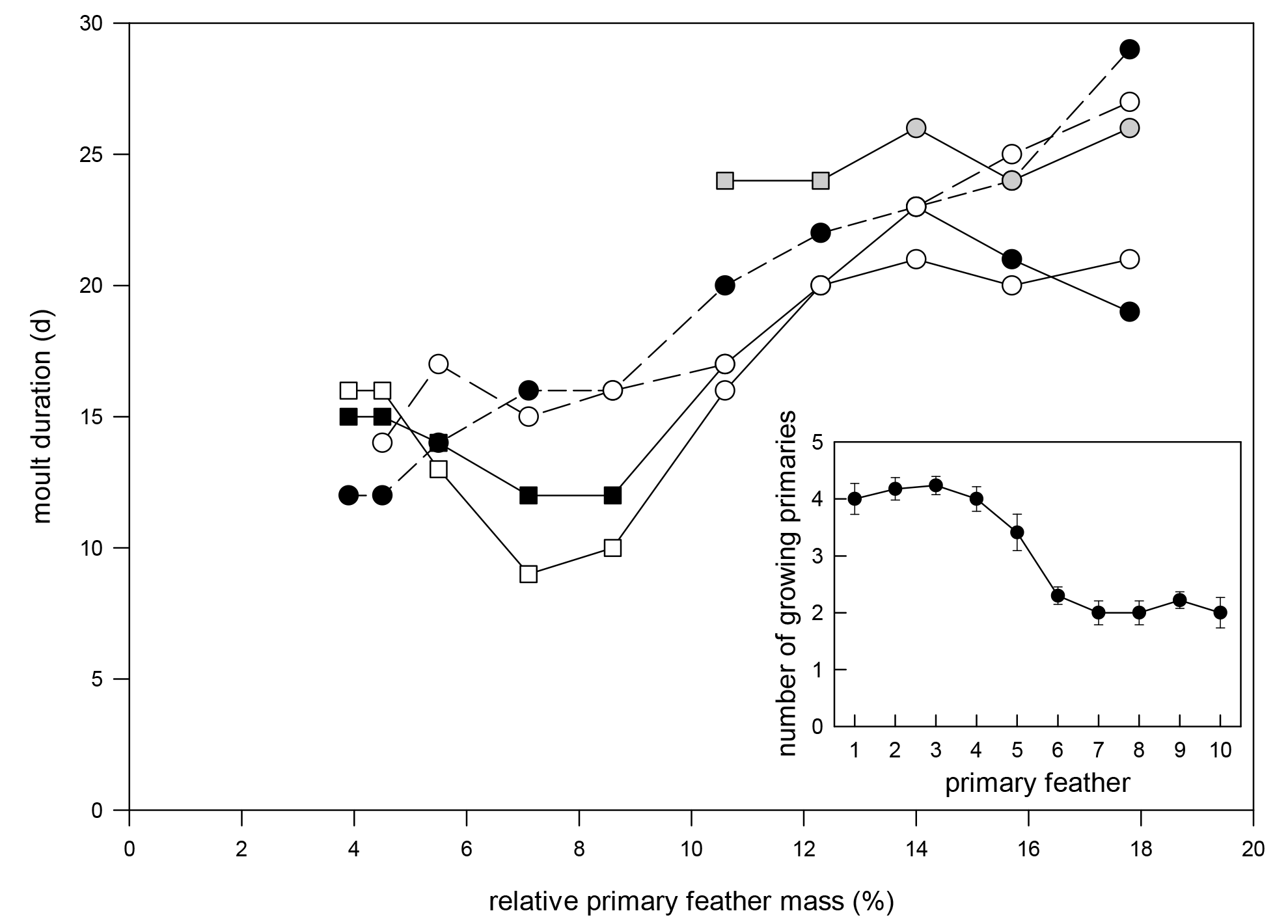

Supplement: Figure S2 — Molt duration of individual primaries versus relative primary feather mass for free-living adult and second-year knots (solid lines) and captive adult red knots (dashed lines). Each point on each curve corresponds to an individual primary as relative primary mass increases with increasing primary number. For adult captive females, the models did not converse to a significant solution for primaries 1 and 7. For second-years we had insufficient data of active molt for primaries 1–5 (Table S3) and data for the sexes were pooled. Closed symbols, males; open symbols, females; grey symbols, second-years; circle, Type 2 model; square, Type 4 model. The inset graph shows the number of simultaneously growing primaries (mean ± SE) for each primary in molt for the average free-living adult knot. (TIF) [file pone.0053890.s002.tif]

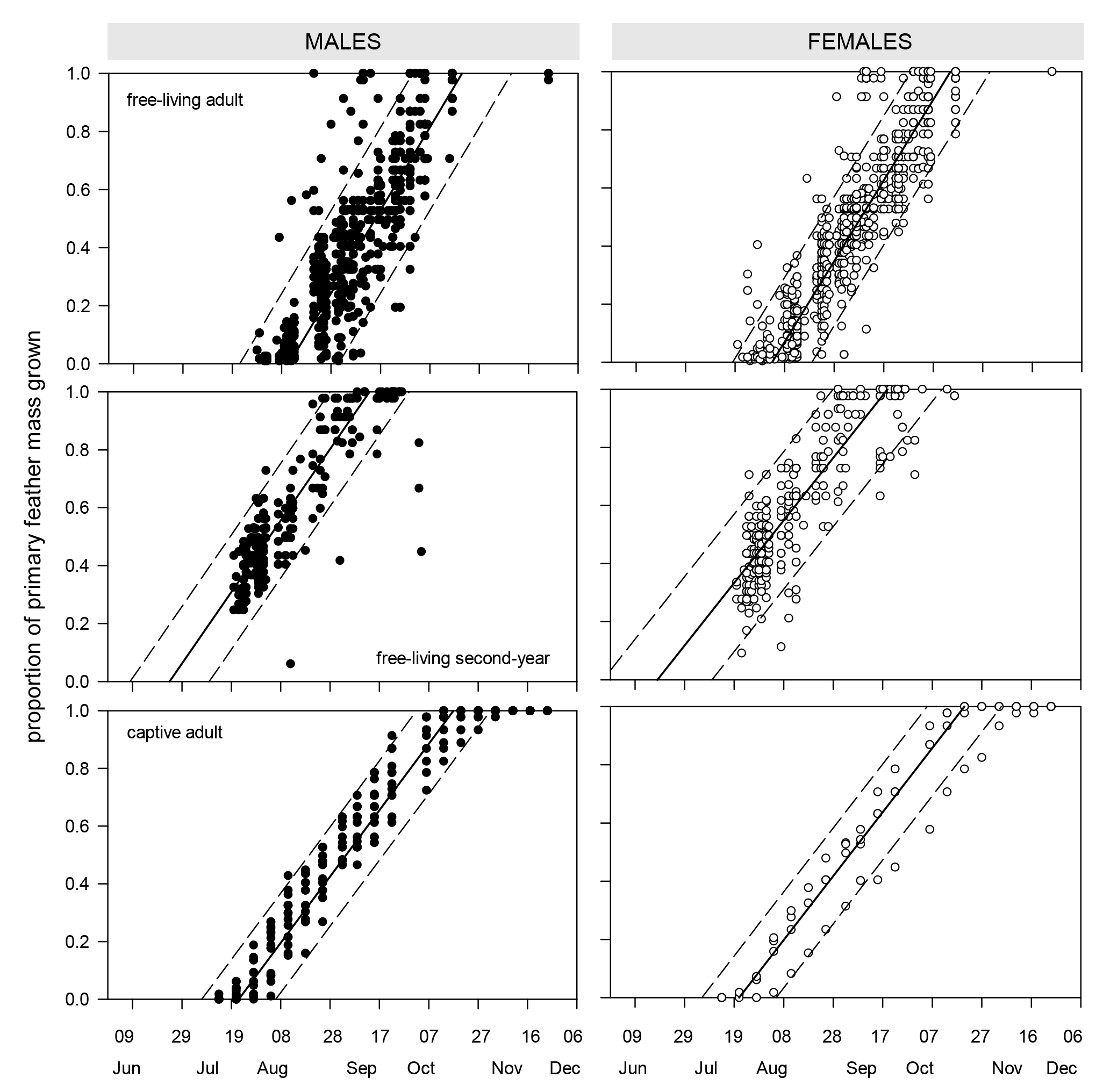

Supplement: Figure S3 — The relationship between the proportion of primary feather mass grown and time of the year for free-living adult and second-year red knots, and captive adult red knots. Left panels, males, closed symbols; right panels, females, open symbols. Solid lines represent the general molt models, dashed lines give the 95% confidence intervals. (TIF) [file pone.0053890.s003.tif]
