# Supplementary material for: When the Seasons Don't Fit: Speedy Molt as a Routine Carry-Over Cost of Reproduction
Source: PLoS One. 2013 Jan 17;8(1):e53890. doi: 10.1371/journal.pone.0053890 (PMC3547963; doi:10.1371/journal.pone.0053890)
Supplement: Information S1 — Extra Information for Tables S1, S2, S3, and S4 and Figures S1 and S2. Description of the Results of the Individual Primary Molt Analyses. (DOCX) [file pone.0053890.s004.docx]

**Extra Information Tables S1-4 and Figures S1-2**

**Results of the Individual Primary Molt Analyses**

To test if the overall proportion of feather mass grown (PFMG) increased linearly with time, a requirement for the molt models of [1] and [2], we calculated primary molt models for each individual primary and for each age and sex class (Type 2 and Type 4, [1,2]). The molt index for the individual primary models is based on feather growth scores and does not use information on primary masses. In free-living adults, Type 2 duration estimates up to primary 5 were clearly inconsistent with observed phenology, whereas Type 4 estimates were clearly consistent; for the outer primaries, Type 2 estimates were consistent and Type 4 ones inconsistent (Tables S1 and S2). Therefore we used Type 4 parameters for primaries 1 through 5 and Type 2 parameters for primaries 6 and higher to test if PFMG of the average adult bird increased linearly with time (cf. [3,4]). For second-years, only Type 2 parameters were used as all birds were permanently present in the area (Table S3). Also for captive adults individual primary models were calculated (Table S4). For each group, the average cumulative feather growth was calculated for each primary. These results were converted to feather growth scores from which PFMGs were calculated as in [1]. The estimated cumulative PFMG increased linearly with time in all groups (Fig S1), and the data fulfilled the requirements of the general model.

The results of the individual primary molt models could also be used to investigate how overall molt duration differences were established. Primary molt took a shorter time to complete in lighter than in heavier primaries (Fig. S2). In captive adults, molt duration increased with relative primary mass, while in free-living adults molt duration initially decreased during the first half of molt, up to primary 5. During this period the highest number of primaries was growing simultaneously in free-living adults (ca. four; inset graph). Molt duration of the last primaries (9 and 10) was also shorter in free-living adults than in captive adults. However, as the 95%-confidence intervals of molt duration overlapped between free-living and captive adults for each primary, the differences were not significant.

**References**

1. Underhill LG, Zucchini W (1988) A model for avian primary moult. Ibis 130: 358-372. doi: 10.1111/j.1474-919X.1988.tb00993.x.

2. Underhill LG, Zucchini W, Summers RW (1990) A model for avian primary moult-data types based on migration strategies and an example using the redshank *Tringa totanus*. Ibis 132: 118-123. doi: 10.1111/j.1474-919X.1990.tb01024.x.

3. Barshep Y, Minton C, Underhill LG, Remisiewicz M (2011) The primary moult of curlew sandpipers *Calidris ferruginea* in North-western Australia shifts according to breeding success. Ardea 99: 43-51. doi: [10.5253/078.099.0106](http://dx.doi.org/10.5253/078.099.0106).

4. Remisiewicz M, Tree AJ, Underhill LG, Gustowska A, Taylor PB (2009) Extended primary moult as an adaptation of adult wood sandpipers *Tringa glareola* to their freshwater habitats in southern Africa. Ardea 97: 271-280. doi: 10.5253/078.097.0302.
