# Supplementary material for: When the Seasons Don't Fit: Speedy Molt as a Routine Carry-Over Cost of Reproduction
Source: PLoS One. 2013 Jan 17;8(1):e53890. doi: 10.1371/journal.pone.0053890 (PMC3547963; doi:10.1371/journal.pone.0053890)
Supplement: Table S1 — Estimates (with asymptotic standard errors) of individual primary models of Types 2 and 4 for free-living adult male red knots. (DOCX) [file pone.0053890.s005.docx]

**Table S1.** Estimates (with asymptotic standard errors) of individual primary models of Types 2 and 4 for free-living adult male red knots.

|  |  | **Type 2** |  |  | | **Type 4** |  | | **sample size by molt status** | | | | | | |
| --- | --- | --- | --- | --- | --- | --- | --- | --- | --- | --- | --- | --- | --- | --- | --- |
| **primary** | **start** | **SD start** | **duration** | **start** | **SD start** | | | **duration** | **not started** | | | **active** | | **finished** | |
| P1 | 208 ± 2.0 | 13 ± 3.4 | 28 ± 2.0 | 223 ± 3.0 | 11 ± 2.9 | | | 15 ± 2.5 | | 0 | 146 | | 398 | |  |
| P2 | 208 ± 2.0 | 13 ± 3.4 | 28 ± 2.0 | 223 ± 3.0 | 10 ± 2.9 | | | 15 ± 2.5 | | 0 | 146 | | 398 | |  |
| P3 | 211 ± 1.8 | 12 ± 3.3 | 26 ± 1.8 | 224 ± 2.9 | 10 ± 2.8 | | | 14 ± 2.4 | | 6 | 143 | | 395 | |  |
| P4 | 218 ± 1.4 | 12 ± 3.0 | 21 ± 1.5 | 229 ± 3.0 | 10 ± 2.8 | | | 12 ± 2.3 | | 29 | 143 | | 372 | |  |
| P5 | 228 ± 1.0 | 11 ± 2.7 | 18 ± 1.2 | 235 ± 3.8 | 11 ± 2.9 | | | 12 ± 3.3 | | 83 | 158 | | 303 | |  |
| P6 | 235 ± 0.9 | 11 ± 2.7 | 17 ± 1.1 | 242 ± 3.6 | 10 ±3.0 | | | 12 ± 2.8 | | 141 | 158 | | 245 | |  |
| P7 | 244 ± 0.8 | 12 ± 2.7 | 20 ± 1.2 | 237 ± 3.2 | 12 ± 3.5 | | | 27 ± 3.4 | | 226 | 178 | | 140 | |  |
| P8 | 254 ± 0.9 | 13 ± 3.0 | 23 ± 1.5 | 249 ± 4.8 | 15 ± 5.6 | | | 29 ± 5.8 | | 312 | 169 | | 63 | |  |
| P9 | 266 ± 1.1 | 14 ± 3.9 | 21 ± 1.9 | 278 ± 14.8 | 21 ± 12.7 | | | 23 ± 14.0 | | 405 | 108 | | 31 | |  |
| P10 | 279 ± 2.1 | 19 ± 5.9 | 19 ± 2.6 | 349 ± 39 | 9 ± 14.1 | | | 1 ± 7.2 | | 465 | 57 | | 22 | |  |
