# Supplementary material for: When the Seasons Don't Fit: Speedy Molt as a Routine Carry-Over Cost of Reproduction
Source: PLoS One. 2013 Jan 17;8(1):e53890. doi: 10.1371/journal.pone.0053890 (PMC3547963; doi:10.1371/journal.pone.0053890)
Supplement: Table S2 — Estimates (with asymptotic standard errors) of individual primary models of Types 2 and 4 for free-living adult female red knots. (DOCX) [file pone.0053890.s006.docx]

**Table S2.** Estimates (with asymptotic standard errors) of individual primary models of Types 2 and 4 for free-living adult female red knots.

|  |  | **Type 2** | |  | |  | **Type 4** |  | **sample size by molt status** | | | |
| --- | --- | --- | --- | --- | --- | --- | --- | --- | --- | --- | --- | --- |
| **primary** | **start** | | **SD start** | | **duration** | **start** | **SD start** | **duration** | **not started** | **active** | **finished** | |
| P1 | 205 ± 1.1 | | 9 ± 2.0 | | 27 ± 1.4 | 215 ± 1.9 | 8 ± 1.8 | 16 ± 1.9 | 0 | 211 | 559 | |
| P2 | 205 ± 1.1 | | 9 ± 2.0 | | 27 ± 1.3 | 216 ± 1.9 | 8 ± 1.8 | 16 ± 1.9 | 3 | 212 | 555 | |
| P3 | 209 ± 0.9 | | 9 ± 1.9 | | 24 ± 1.1 | 219 ± 1.9 | 7 ± 1.7 | 13 ± 1.8 | 17 | 201 | 552 | |
| P4 | 215 ± 0.8 | | 8 ± 1.9 | | 19 ± 1.0 | 226 ± 2.1 | 6 ± 1.8 | 9 ± 1.8 | 58 | 181 | | 531 |
| P5 | 222 ± 0.7 | | 9 ± 1.9 | | 17 ± 0.9 | 229 ± 1.9 | 7 ± 1.7 | 10 ± 1.8 | 121 | 174 | | 475 |
| P6 | 230 ± 0.6 | | 8 ± 1.8 | | 16 ± 0.9 | 233 ± 1.9 | 7 ± 2.0 | 13 ± 1.9 | 201 | 184 | | 385 |
| P7 | 237 ± 0.6 | | 8 ± 1.7 | | 20 ± 0.9 | 233 ± 1.7 | 9 ± 2.2 | 25 ± 2.0 | 287 | 231 | | 252 |
| P8 | 248 ± 0.6 | | 9 ± 1.9 | | 21 ± 1.0 | 247 ± 2.2 | 10 ± 2.4 | 22 ± 2.4 | 401 | 214 | | 155 |
| P9 | 260 ± 0.8 | | 12 ± 2.7 | | 20 ± 1.2 | 251 ± 5.0 | 17 ± 6.4 | 32 ± 6.0 | 531 | 168 | | 71 |
| P10 | 269 ± 1.0 | | 14 ± 3.6 | | 21 ± 1.7 | 263 ± 18.5 | 42 ± 28.7 | 62 ± 32.2 | 602 | 138 | | 30 |
