# Supplementary material for: When the Seasons Don't Fit: Speedy Molt as a Routine Carry-Over Cost of Reproduction
Source: PLoS One. 2013 Jan 17;8(1):e53890. doi: 10.1371/journal.pone.0053890 (PMC3547963; doi:10.1371/journal.pone.0053890)
Supplement: Table S3 — Estimates (with asymptotic standard errors) of individual primary models of Types 2 and 4 for free-living second-year red knots (sexes combined). (DOCX) [file pone.0053890.s007.docx]

**Table S3.** Estimates (with asymptotic standard errors) of individual primary models of Types 2 and 4 for free-living second-years red knots (sexes combined).

|  |  | **Type 2** | |  | | **sample size by molt status** | | |
| --- | --- | --- | --- | --- | --- | --- | --- | --- |
| **primary** | **start** | | **SD start** | | **duration** | **not started** | **active** | **finished** |
| P6 | 187 ± 2.3 | | 10 ± 3.1 | | 27 ± 2.5 | 5 | 213 | 368 |
| P7 | 201 ± 0.9 | | 11 ± 2.5 | | 26 ± 1.5 | 67 | 282 | 237 |
| P8 | 214 ± 0.7 | | 11 ± 1.2 | | 26 ± 2.3 | 225 | 203 | 158 |
| P9 | 226 ± 0.9 | | 12 ± 2.6 | | 24 ± 1.3 | 340 | 135 | 111 |
| P10 | 236 ± 1.1 | | 13 ± 2.9 | | 26 ± 1.6 | 403 | 133 | 50 |

Note: For P1 to P5 no or insufficient data on active molt was available.
