# Supplementary material for: When the Seasons Don't Fit: Speedy Molt as a Routine Carry-Over Cost of Reproduction
Source: PLoS One. 2013 Jan 17;8(1):e53890. doi: 10.1371/journal.pone.0053890 (PMC3547963; doi:10.1371/journal.pone.0053890)
Supplement: Table S4 — Estimates (with asymptotic standard errors) of individual primary models of Types 2 and 4 for captive adult male and female red knots. (DOCX) [file pone.0053890.s008.docx]

**Table S4.** Estimates (with asymptotic standard errors) of individual primary models of Type 2 for captive adult male and female red knots.

|  |  | **male** |  | **sample size by molt status** | | |  | **female** | |  | | **sample size by molt status** | | |
| --- | --- | --- | --- | --- | --- | --- | --- | --- | --- | --- | --- | --- | --- | --- |
| **primary** | **start** | **SD start** | **duration** | **not started** | **active** | **finished** | **start** | | **SD start** | | **duration** | **not started** | **active** | **finished** |
| P1 | 201 ± 2.4 | 24 ± 6.9 | 12 ± 2.1 | 116 | 27 | 323 | -- | | -- | | -- | 37 | 8 | 105 |
| P2 | 202 ± 2.4 | 24 ± 7.0 | 12 ± 2.1 | 117 | 28 | 321 | 202 ± 2.6 | | 10 ± 4.2 | | 14 ± 3.2 | 37 | 12 | 101 |
| P3 | 203 ± 2.4 | 24 ± 6.8 | 14 ± 2.2 | 120 | 33 | 313 | 205 ± 2.6 | | 10 ± 4.0 | | 17 ± 3.3 | 40 | 14 | 96 |
| P4 | 210 ± 2.4 | 25 ± 6.8 | 16 ± 2.4 | 134 | 37 | 295 | 214 ± 3.0 | | 14 ± 5.5 | | 15 ± 3.5 | 47 | 13 | 90 |
| P5 | 218 ± 2.6 | 27 ± 7.5 | 16 ± 2.4 | 152 | 36 | 278 | 222 ± 3.4 | | 19 ± 6.9 | | 16 ± 3.8 | 54 | 13 | 83 |
| P6 | 225 ± 2.7 | 31 ± 8.1 | 20 ± 2.7 | 169 | 45 | 252 | 231 ± 3.8 | | 22 ± 7.8 | | 17 ± 4.0 | 61 | 14 | 75 |
| P7 | 237 ± 2.8 | 33 ± 8.5 | 22 ± 2.8 | 196 | 49 | 221 | -- | | -- | | -- | 70 | 18 | 62 |
| P8 | 252 ± 2.9 | 35 ± 8.9 | 23 ± 2.9 | 229 | 20 | 187 | 255 ± 4.3 | | 27 ± 9.4 | | 23 ± 4.8 | 80 | 18 | 52 |
| P9 | 265 ± 2.9 | 34 ± 8.8 | 24 ± 3.0 | 257 | 20 | 159 | 270 ± 4.6 | | 30 ± 10.4 | | 25 ± 5.1 | 92 | 18 | 40 |
| P10 | 280 ± 3.2 | 38 ± 9.9 | 29 ± 3.3 | 287 | 59 | 120 | 284 ± 4.9 | | 31 ± 11.1 | | 27 ± 5.5 | 101 | 19 | 30 |

Note: The models for primaries 1 and 7 for female knots did not converge to a significant solution.
