# Supplementary material for: When the Seasons Don't Fit: Speedy Molt as a Routine Carry-Over Cost of Reproduction
Source: PLoS One. 2013 Jan 17;8(1):e53890. doi: 10.1371/journal.pone.0053890 (PMC3547963; doi:10.1371/journal.pone.0053890)
Supplement: Table S5 — Body mass and body size characteristics of free-living second-year and adult red knots that had completed primary molt. (DOCX) [file pone.0053890.s009.docx]

**Table S5.** Body mass and body size characteristics of free-living second-year and adult red knots that had completed primary molt.

|  | **body mass (g)** | **wing length (mm)** | **bill length (mm)** | **tarsus length (mm)** |
| --- | --- | --- | --- | --- |
| adult male | 139 ± 1.6 (18)^a^ | 170 ± 0.8 (13)^a,b^ | 32.7 ± 0.3 (18)^a^ | 31.8 ± 0.3 (18) |
| adult female | 150 ± 2.1 (25)^b^ | 175 ± 0.5 (23)^c^ | 34.3 ± 0.3 (25)^b^ | 32.1 ± 0.3 (25) |
| second-year male | 138 ± 1.6 (24)^a^ | 169 ± 0.8 (23)^a^ | 32.6 ± 0.4 (25)^a^ | 31.2 ± 0.2 (25) |
| second-year female | 149 ± 1.8 (23)^b^ | 173 ± 0.9 (24)^b,c^ | 34.1 ± 0.4 (25)^b^ | 32.2 ± 0.3 (25) |

Note: Sample size is given in parentheses. Not all variables were always determined and occasionally an erroneous number was noted. Groups that differed significantly from each other within a column have different alphabetical subscripts (ANOVA, Tukey post-hoc analysis).
