# Supplementary material for: When the Seasons Don't Fit: Speedy Molt as a Routine Carry-Over Cost of Reproduction
Source: PLoS One. 2013 Jan 17;8(1):e53890. doi: 10.1371/journal.pone.0053890 (PMC3547963; doi:10.1371/journal.pone.0053890)
Supplement: Table S6 — Final estimates (with asymptotic standard errors for free-living red knots and SE for captive knots) of general molt parameters. (DOCX) [file pone.0053890.s010.docx]

**Table S6.** Final estimates (with asymptotic standard errors for free-living red knots and SE for captive knots) of general molt parameters.

|  | **molt parameters** | | | | **sample size by molt status** | | | **range individual molt models** | | | | |
| --- | --- | --- | --- | --- | --- | --- | --- | --- | --- | --- | --- | --- |
| **group** | **start** | **SD start** | **duration** | **end** | **none** | **active** | **finished** | **start min** | **start max** | | **duration min** | **duration max** |
| SYM | 175 ± 1.3 | 8 ± 1.6 | 81 ± 2.1 | 256 | 0 | 233 | 25 |  |  |  | |  |
| SYF | 169 ± 1.8 | 11 ± 2.4 | 93 ± 2.9 | 262 | 0 | 294 | 25 |  |  |  | |  |
| FAdM | 223 ± 1.1 | 10 ± 2.0 | 70 ± 2.1 | 293 | 0 | 492 | 18 |  |  |  | |  |
| FAdF | 215 ± 0.7 | 8 ± 1.4 | 72 ± 1.3 | 287 | 0 | 649 | 25 |  |  |  | |  |
| CAdM | 203 ± 1.6 | 7.5 | 87 ± 1.5 | 290 ± 1.9 | 55 | 240 | 144 | 191 | 212 | 75 | | 100 |
| CAdF | 202 ± 2.3 | 7.8 | 93 ± 2.3 | 295 ± 4.7 | 27 | 137 | 70 | 192 | 213 | 80 | | 107 |
| CAdMf | 201 ± 1.6 | 5.7 | 85 ± 2.2 | 289 ± 2.5 | 19 | 156 | 72 | 194 | 212 | 75 | | 100 |
| CAdFf | 204 ± 3.5 | 6.9 | 92 ± 4.2 | 296 ± 7.4 | 6 | 52 | 18 | 199 | 214 | 85 | | 104 |

Note: A Type 4 model was used for free-living adult red knots and a Type 2 model for second-years. For captive knots, the results are the means of the individual molt models. Range of the start and duration of individual bird models of the captive birds is given in the last columns. Start and end date are given in Julian day. Group abbreviations: SYM, second-year males; SYF, second-year females; FAdM, free-living adult males; FAdF, free-living adult females; CAdM, captive adult males 2009; CAdF, captive adult females 2009; CAdMf, captive adult males first molt; CAdFf, captive adult females first molt
